# Supplementary material for: Demographic, social, and behavioral correlates of SARS-CoV-2 seropositivity in a representative, population-based study of Minnesota residents
Source: PLoS One. 2023 Jun 15;18(6):e0279660. doi: 10.1371/journal.pone.0279660 (PMC10270347; doi:10.1371/journal.pone.0279660)
Supplement: Attachment — Submitted filename: Response to Reviewers PONE-D-22-04448 FINAL.docx [file pone.0279660.s003.docx]

We thank the reviewers for their thoughtful comments and suggestions. We have attempted to be as responsive as possible to each comment, our response is detailed below. Line numbers refer to the “clean” version of the document with changes accepted (Manuscript PONE-D-22-0448).

| **Editor Comments** | **Author Response** |
| --- | --- |
| Please ensure that your manuscript meets PLOS ONE's style requirements, including those for file naming. | We have verified that our manuscript meets the formatting guidelines and have adjusted as necessary |
| Please amend your current ethics statement to address the following concerns: a) Did participants provide their written or verbal informed consent to participate in this study? b) If consent was verbal, please explain i) why written consent was not obtained, ii) how you documented participant consent, and iii) whether the ethics committees/IRB approved this consent procedure. | Informed consent was obtained electronically within the survey for participants in the COVID-19 Household Impact Survey (CIS). Participants in the MCAS were provided a link to the study portal and a unique ID where they can read more about the study, provide their consent to receive a capillary blood collection kit, and provide an address and a phone number to send the kit. Consent was active (and assumed to be “written” in the ethics statement parlance) and secured via the following language:  “The survey should take no more than 10 minutes to complete and participation is voluntary. When you have completed each page of the survey, click on the “NEXT” button at the bottom of each page. If you want to stop the survey at any time, click the SAVE AND EXIT button at the top of the page. After the survey, you will receive a $10 gift code as a thank you for your participation.  Your participation in this survey is voluntary and we will make every effort to keep your responses confidential. The information you provide will be used for research purposes only and will not be shared with the government. Only select members of the research team will have access to your responses. There are no personal benefits to participating in this study, and risks to participating in this study are minimal. However, if any of the questions make you uncomfortable, you may skip them as well as any you are unable to answer. You may also choose to stop at any time.  If you have any questions about the study, you can contact us toll free 1-877-393-9734 or by email at mncovid19@norc.org  By continuing to the next screen, you agree to participate in the survey and are confirming your name is [P_NAME]. If you are not this person, please do not continue. Thank you.”  A fuller consent form that was sent to prospective respondents in advance is appended to this document.  We have added the written consent line to lines 164-166 of the revised manuscript, indicating that the University of Minnesota Institutional Review Board reviewed and approved this consent procedure. |
| Please state what role the funders took in the study. | We wish to update this language and have done so below and in the cover letter:  “Role of Funder: The study design was completed in advance of funding receipt and was the basis of the awarded contract. Data analysis and manuscript preparation was performed by the author team, which includes a scientist employed by the funder, the Minnesota Department of Health (Dr. Stephanie Yendell), who consulted on epidemiology and state infection trends. The funder (MDH) had no role in the decision to publish.” |
| Please remove any funding-related text from the manuscript and let us know how you would like to update your Funding Statement. | We have removed the funding-related text from the manuscript and described how we would like to cast the funding statement and role of the funder (per the above response) in the cover letter. |
| We note that you have stated that you will provide repository information for your data at acceptance. Should your manuscript be accepted for publication, we will hold it until you provide the relevant accession numbers or DOIs necessary to access your data. If you wish to make changes to your Data Availability statement, please describe these changes in your cover letter and we will update your Data Availability statement to reflect the information you provide. | We do not wish to make any changes to our Data Availability Statement. |
| Please include a copy of Table 13 which you refer to in your text on page 14. | The text in question was meant to reference table 2, and we have updated the manuscript to correct this error. |
| **Reviewer Comments** | **Author Response** |
| **Reviewer 1** | |
| Was mass immunization initiated in Minnesota during the period prior to or during the collection of the seroprevalence samples? (If not, this could simply be stated in the paper. If so, how would this affect the results of antibody tests? Were any questions asked regarding immunization status prior to sampling?) | During the period of our study, Minnesota had not yet begun mass public vaccination. Vaccine distribution began on December 16, 2020, and was limited to healthcare workers and those in long-term care facilities under Phase 1a of Minnesota’s vaccine rollout plan until January 19, 2021 when Phase 1b began (per historic press releases). Phase 1b expanded access to high risk populations and essential workers and lasted until March 9, 2021. At that point, Phase 1c began, which expanded access within the same categories. Phase 2 began on March 30, 2021, and expanded access to the general public over aged 16.  Our collection period overlapped with Phase 1a and a portion of Phase 1b. Residents of long-term care facilities, who were among the 1a target populations, were not part of our address-based sample collection. However, it is possible that some healthcare workers, essential workers, or high risk individuals in our sample did receive one or both of the first vaccine doses prior to providing a sample for seropositivity testing in our study.  Respondents were not asked about their vaccination status, but the impact of such individuals on our results is likely to be small. The most likely individuals in our sample to have received a vaccine is the 65 and older group. Our sample collection concluded on February 26, 2021. At that point, 21% percent of the 16+ population in Minnesota had received at least one vaccine dose. Individuals aged 65+ made up a little under half the immunized population at that point. These numbers are markedly lower at earlier points in our sample collection, e.g December 31, 2020, and January 31, 2021. Moreover, seropositivity has been shown to be extremely low among individuals who have received only one vaccine dose, which would have been the case for many in our sample^1^. [[NEJM letter (NEJMc2101667) linked below in Additional Citations.](https://www.nejm.org/doi/full/10.1056/NEJMc2101667)]  Because of their higher vaccination rates, individuals over 65 may show seropositivity as a result of vaccination status rather than infection. This indicates some caution is warranted in interpreting the positive association with older age and seropositivity as stemming from infection. We have modified the text at lines 243-254 to address this.  “Third, COVID-19 vaccines were being made available to healthcare workers and other high priority groups during the blood sample collection phase of the study. Respondents did not report whether or not they had been vaccinated when returning their samples, so it is not possible to control for or adjust our samples for potential vaccination. However, the impact of early vaccine access on our results is likely to be small, since access was highly restricted during this period and many individuals with vaccine access had only received one dose, which has been shown to be unlikely to generate a seropositive result. The one exception is the population 65 and older, where access had risen such that a quarter of the population had completed the 2-dose vaccine series by the end of our collection period, up from 0.3% at the collection midpoint. We therefore interpret the positive association between older age and seropositivity in our results with some caution, as this is the one dimension in which vaccine access may have generated positivity, in addition to infection.”  In terms of our other two qualitative results, vaccination is unlikely to affect our interpretation. Individuals with school-aged children are not likely to be in groups with vaccine access during our study period. Individuals exhibiting more protective behaviors may have adopted vaccines early, if eligible, but this means our estimate of infection reduction associated with these behaviors is, if anything, an underestimate. |
| What was the rationale for the division of age categories into 18-22, 23-64 and >65? Do these age groups differ significantly in terms of social mobility (for example is >65 the conventional "retirement age"?) and hence in terms of risk of exposure to COVID-19? Would further subdivisions within the second of these categories (say 23-44 and 45-64) have allowed a more precise analysis of the data? | We agree that more granular age categories could better capture life course circumstances associated with COVID-19 risk, and we also agree that the number of respondents under aged 22 in our sample is small. We implemented our three age bins primarily because we had insufficient numbers of respondents at finer age groupings, and the three chosen grouping reflect individuals’ relationship to the labor force, where exposure during work is a concern. As noted in the response to question 1, separately identifying the 65 and older population with an age bin is important because this is the one group that may have had early vaccine access during our sample period. We therefore believe it is important to identify seropositivity for this group separately, as we do with the coefficient for this group in our multivariate logit regression. |
| Were any of the study participants employed in a field that entails greater exposure to COVID-19? (This would include not just healthcare workers but other "frontline" workers, such as law enforcement officers, or those involved in the service industries). This would constitute an independent risk factor for SARS-CoV-2 infection; if information on this is available, it could be included in the analyses. | We thank the reviewer for this question, and agree that occupation had very meaningful implications for seroprevalence risk, particularly in the first year of the pandemic. Unfortunately, CIS data not ask about specific occupation. We have updated our limitations section at lines 257-261 to reflect the lack of occupational data:  “Sixth, despite extensive questions on demographic and behavioral COVID-19 risk factors, some factors that could meaningfully impact seroprevalence including substance use before and during the pandemic and occupation in high-risk fields (e.g. healthcare and service workers) were not asked about in CIS.” |
| Was any information collected on substance use (alcohol, tobacco, etc.) by study participants? (If not, this could be mentioned among the limitations of the study, as this may constitute a risk factor for infection, and also because there are several reports of increased substance use during periods of isolation or "lockdown"). | Personal substance use was not asked about during the COVID-19 Household Impact Survey. We have updated our limitations section with the below text at lines 257-261 to reflect this:  “Sixth, despite extensive questions on demographic and behavioral COVID-19 risk factors, some factors that could meaningfully impact seroprevalence including substance use before and during the pandemic and occupation in high-risk fields (e.g. healthcare and service workers) were not asked about in CIS.” |
| What was the epidemiological status of the concerned state / region at the time the study was conducted? Was sampling done during a local "wave" or "spike" of SARS-CoV-2 cases, or during a period of relative quiescence? This could affect the interpretation of the study results. Even if a formal statistical analysis is not carried out, some basic epidemiological details could be provided based on data from local health authorities. | We appreciate the reviewer’s request for additional context on community seropositivity trends in the location of our study. During the six months between the end of survey collection and the beginning of blood sample collection (June – Dec 2020), Minnesota experienced a spike in COVID cases that peaked in November 2020. This paralleled the fall 2020 wave experienced in many parts of the US.  We have updated our discussion section at lines 201-207 to better reflect observed community prevalence in Minnesota:  “The MCAS study links demographic and behavioral data from early in the COVID-19 pandemic to serostatus six months later. During this 6-month period, the State of Minnesota reached a peak reported seropositivity rate of 17.2% in November 2020. The overall MCAS seropositivity rate (11.81%) was lower than the estimated seroprevalence of 15.9% (95% CI, 13.3%-18.6%) observed for Minnesota in the CDC nationwide commercial laboratory survey in the first half January 2021. During this 6-month period, Minnesota also experienced rises in hospitalizations and deaths that paralleled the experiences of many other parts of the US.” |
| Did any study participants develop overt COVID-19 (diagnosed via antigen testing or RT-PCR) in the period between baseline data collection and subsequent antibody testing? This might be of interest when analyzing the frequency of symptomatic vs asymptomatic infection and their respective correlates (for example, was asymptomatic infection more common in younger subjects, or symptomatic infection more common in the elderly?) | We agree that information on laboratory confirmed COVID cases detected during the window between the baseline and endpoint of our study would allow for interesting and important additional analysis, particularly as regards to asymptomatic COVID-19. Unfortunately, no data were collected on COVID-19 diagnosis between survey and bio-sample collection. We have updated our limitations at lines 254-256 to reflect this:  “Fourth, respondents did not report whether they had been formally diagnosed with COVID-19 between the baseline survey and bio-sample collection, so we cannot distinguish between previously detected and unreported cases.” |
| The Discussion section could be improved by a mention of the age-, income- and location-related differences identified in this study. Though they may be seen as a replication of earlier results, these findings remain of value in terms of larger-scale social and public health interventions to minimize the risk of infection (e.g. by providing financial assistance or minimizing overcrowding in housing). | We have added text to our discussion at lines 208-211 to reflect this:  “First, our multi-variate regression found that older age groups were associated with higher odds of seropositivity and that living in a higher income household was associated lower odds of seropositivity. These findings further demonstrate socio-economic disparities in COVID-19 risk.^33,34^” |
| The finding regarding school-age children is interesting, but it cannot be taken as definitive without a knowledge of the children's antibody / antigen status. There are additional explanations that need to be taken into account (e.g. an association between children in the house and lower income; greater potential exposure to infection by parents / caregivers due to the need to purchase essential goods for their children). The wording regarding this finding could be "softened" a little and the need for replication (and for the exclusion of alternate explanations) could be discussed towards the end of the paper. | We agree that the presence of school-aged children in the home can indeed co-vary with other potential explanatory pathways. We note that our multivariate analysis goes some way toward addressing that concern. For example, although families with school-aged children do tend to be lower income, our multivariate logit adjusts for this along with a number of other potential confounders. However, we have updated our discussion at lines 213-224 to better reflect the conclusions that can be drawn from our study design:  “The significance and magnitude of the association between seropositivity and living with children of school age suggest that there are COVID-19 risk factors associated with the circumstances that accompany raising children. This may support the fact that school-age children can become infected and transmit SARS-CoV-2 infections and contribute to family and community spread; however, our observational findings cannot determine whether this is the specific driving force of our findings, or some other feature associated with having school-aged children made respondents to the survey more vulnerable to the spread of COVID-19. Further research should be conducted to determine if school-aged children are meaningful contributors to transmission, to inform whether broader testing efforts in schools might be a useful tool to identify infectious individuals, prevent outbreaks, and protect vulnerable members of the community.” |
| What was the reported sensitivity / specificity of the antibody tests themselves? Was this data available, and how would it affect the confidence which one could place in the final study results? | We thank the reviewer for asking about the limitations of the antibody assay used in this study. This has important implications for the interpretation of our findings, and we have updated our limitations at line 261-264 to reflect this:  “Seventh, we assumed all positive and negative COVID-19 cases as being true cases. In reality, the Human IgG (4-Plex) assay has a reported sensitivity of 97% and specificity of 100%30. This imperfect sensitivity along with our long study window may be biasing our seroprevalence estimates towards a lower value.” |
| **Reviewer 2** | |
| The time period between elicited behavioral and social factors and the infection test is quite long, almost one year. Could the authors cite some sources that show how these factors have been relatively stable over time, even though the research findings and the respective policies had changed quite substantially over this same time period? | We are unaware of any research validating the stability of these measures over time and acknowledge this as a limitation at line 261 (text quoted below). Involved authors are currently working with a second wave of data from this cohort, and these analyses will hopefully produce a better understanding of how these behavioral and social factors evolved over the course of the pandemic.  “Finally, we acknowledge that many of the variables, such as symptoms, mental health, and personal and public health mitigation behaviors likely varied between the time of the initial survey and the subsequent blood specimen collection. We are in the process of fielding a follow-up study that deploys a design that supports a more contemporaneous assessment.” |
| Although the range of the elicited behavioral covariates is substantive, it appears that one important variable is not controlled for, namely whether the participants had already been tested positive before and/or whether they had been vaccinated. (Or perhaps I missed it and these people were excluded from the study?) Having been tested positive or getting vaccinated could have behavioral effects, e.g. engaging in fewer mitigating activities due to the newly gained (temporary) immunity. | Thank you for this comment. There are several points, and we address them in turn. We view a prior Covid infection and vaccination as separate concerns. COVID-19 diagnosis was asked about during the initial survey (March – June 2020), but it was very rarely reported (fewer than 5 cases in our sample), and we chose not include this indicator in our model due to the small sample size.  Regarding vaccine status, During the period of our study, Minnesota had not yet begun mass public vaccination. Vaccine distribution began on December 16, 2020, and was limited to healthcare workers and those in long-term care facilities under Phase 1a of Minnesota’s vaccine rollout plan until January 19, 2021 when Phase 1b began (per historic press releases). Phase 1b expanded access to high risk populations and essential workers and lasted until March 9, 2021. At that point, Phase 1c began, which expanded access within the same categories. Phase 2 began on March 30, 2021, and expanded access to the general public over aged 16.  Our collection period overlapped with Phase 1a and a portion of Phase 1b. Residents of long-term care facilities, who were among the 1a target populations, were not part of our address-based sample collection. However, it is possible that some healthcare workers, essential workers, or high risk individuals in our sample did receive one or both of the first vaccine doses prior to providing a sample for seropositivity testing in our study.  Respondents were not asked about their vaccination status, but the impact of such individuals on our results is likely to be small. The most likely individuals in our sample to have received a vaccine is the 65 and older group. Our sample collection concluded on February 26, 2021. At that point, 21% percent of the 16+ population in Minnesota had received at least one vaccine dose. Individuals aged 65+ made up a little under half the immunized population at that point. These numbers are markedly lower at earlier points in our sample collection, e.g December 31, 2020, and January 31, 2021. Moreover, seropositivity has been shown to be extremely low among individuals who have received only one vaccine dose, which would have been the case for many in our sample. [NEJM letter added to references section, #35]  Because of their higher vaccination rates, individuals over 65 may show seropositivity as a result of vaccination status rather than infection. This indicates some caution is warranted in interpreting the positive association with older age and seropositivity as stemming from infection. We have modified the text at lines 240-251 to address this.  In terms of our other two qualitative results, vaccination is unlikely to affect our interpretation. Individuals with school-aged children are not likely to be in groups with vaccine access during our study period. Individuals exhibiting more protective behaviors may have adopted vaccines early, if eligible, but this means our estimate of infection reduction associated with these behaviors is, if anything, an underestimate.  Finally, we thank the reviewer for raising the importance of known COVID-19 diagnosis. Unfortunately, COVID-19 and vaccination statuses were not asked about during blood sample collection . COVID-109 diagnosis was asked about during the initial survey (March – June 2020), but it was very rarely reported (fewer than 5 cases), and we chose not include this indicator in our model due to the general lack of availability of COVID 19 testing early in the pandemic. We have addressed the lack of COVID diagnosis and vaccination data in our limitations section starting at lines 243-254:  “Third, COVID-19 vaccines were being made available to healthcare workers and other high priority groups during the blood sample collection phase of the study. Respondents did not report whether or not they had been vaccinated when returning their samples, so it is not possible to control for or adjust our samples for potential vaccination. However, the impact of early vaccine access on our results is likely to be small, since access was highly restricted during this period and many individuals with vaccine access had only received one dose, which has been shown to be unlikely to generate a seropositive result [NEJM]. The one exception is the population 65 and older, where access had risen such that a quarter of the population had completed the 2-dose vaccine series by the end of our collection period, up from 0.3% at the collection midpoint. We therefore interpret the positive association between older age and seropositivity in our results with some caution, as this is the one dimension in which vaccine access may have generated positivity, in addition to infection.” |
| The authors mention that similar datasets were gathered for U.S. adult household population nationwide and for 18 regional areas including 10 states and 8 Metropolitan Statistical Areas. If possible, could the authors mention to what extent their Minnesota-specific results replicate elsewhere in the U.S.? | We thank the reviewer for their comment on the National scope of the CIS survey and how past work compares to our findings. Several papers have used CIS data to examine COVID related behaviors, but ours is the first to our knowledge that links data from this representative survey to seroprevalence data. It is therefore not possible to say at this time whether results from our representative population of Minnesota residents generalize to other US populations. |
| Abstract & Introduction: This following paragraph in the abstract is a bit difficult to read and interpret. Please reconsider whether it does justice to your results in the summary, or whether perhaps it is more suited for the detailed results section and a more intuitively worded results summary could be drafted instead.  “In adjusted multivariate logistic regression models, significant associations between seroprevalence and the following were observed; participants’ age categories (17.794 [1.217-260.083] for 23-64 years and 24.682 [1.506-404.440] for 65+ years compared to the 18-22 years category); income category (0.203 [0.045-0.914] for $30k to <$60k, 0.141 [0.033- 0.604] for $60k to <$125k, and 0.137 [0.024-0.763] for $125k+ compared to the <$30k category); and a binary variable for the presence of household member(s) in the age range of 6 to 17 years old (8.253 [1.195-56.984] compared to those who did not have household member(s) in this age group).» | We have reworded it to make it more readable (see lines 62-71):  “In adjusted multivariate logistic regression models, significant associations between seroprevalence and the following were observed; being from 23-64 and 65+ age groups were both associated with higher odds of COVID-19 seropositivity compared to the 18-22 age group (17.8 [1.2-260.1] and 24.7 [1.5-404.4] respectively). When compared to a less than $30k annual income reference group, all higher income groups had significantly lower odds of seropositivity. Reporting practicing a number of 10 (median reported value in sample) or more of 19 potential COVID-19 mitigation factors (e.g. handwashing and mask wearing) was associated with lower odds of seropositivity (0.4 [0.1-0.99]) Finally, the presence of at least one household member in the age range of 6 to 17 years old was associated with higher odds of seropositivity (8.3 [1.2-57.0]).” |
| Abstract: Please consider introducing a brief definition of the “mitigation score” in the abstract already. | We have included the following text in the abstract to explain the mitigation score (see lines 67-69):  “Reporting practicing a number of 10 (median reported value in sample) or more of 19 potential COVID-19 mitigation factors (e.g. handwashing and mask wearing)” |
| Methods: It is somewhat unclear how the various variables of interest were chosen to be included in the study, i.e. what is the theoretical basis and/or previous evidence for each of them. | Due to how early in the pandemic our data was collected, there was little non-anecdotal evidence on COVID risk factors, and our study was largely exploratory and driven by the questions NORC chose to ask in the CIS survey. |
| Methods: Is it possible to say to what extent do the results hold if slightly different approaches to collapsing the variables are chosen? It appears that some definitions of the variables differ from the ones described in Swaziek et al. (2020) and Wozniak (2020). For example, it is not intuitively clear why the 3 very different age categories (18-22 with only 13(!) participants, 23-64 with 369 participants, older than 64 with 155 participants) are used. Interpretation of these variables can thus be somewhat problematic, as also potentially demonstrated by the differing significance levels between the two logit models presented in Table 2. | We thank the reviewer for their question regarding how we have determined categorical variable groupings. We agree that more granular age categories could better capture life course circumstances associated with COVID-19 risk, and we also agree that the number of respondents under aged 22 in our sample is small. We implemented our three age bins primarily because we had insufficient numbers of respondents at finer age groupings, and the three chosen grouping reflect individuals’ relationship to the labor force, where exposure during work is a concern. As noted in the response to question 1, separately identifying the 65 and older population with an age bin is important because this is the one group that may have had early vaccine access during our sample period. We therefore believe it is important to identify seropositivity for this group separately, as we do with the coefficient for this group in our multivariate logit regression. Additionally, unlike Swaziek et al. (2020) and Wozniak (2020), we linked CIS survey data to seropositivity data. This linkage resulted in smaller sample sizes and a more sensitive data product. Our data use agreement mandated that we use the more collapsed, 3 level age category variable which was created for us my NORC. |
| Methods: Would it be possible to include more information about the extent to which the variables described in the “Study design and population” and “Study variables” subsections differ and the reasons behind this difference? For example, it is not clear how the mitigation behaviors (as measured by the Big 2 and median of 10 (other?) mitigation behaviors) are related to the data collected on “nineteen mitigation behaviors” mentioned earlier. In addition, it would be interesting to see which of the mitigation behaviors “work better” for preventing infections than others, rather than just examining the absolute number of these behaviors. In fact, the paper does mention this in passing in the Discussion: “While not all of these mitigation measures directly affected one’s likelihood of infection, this metric seems to have captured a more general attitude. | There were 19 total factors and the observed median number of reported factors was 10. We have edited our data section at lines 122-129 to make this clearer and explain that the “Big 2” separately represents specific behaviors that have been shown to actually reduce risk:  “The CIS survey asked about 19 behaviors that people may engage in to reduce their risk of acquiring COVID-19. We incorporated these questions into our analysis in two ways. First, we dichotomized the total count of reported behaviors around the sample median of 10 reported behaviors. We also examined if respondents engaged in the specific behaviors of masking and social distancing, which have since been shown to meaningfully reduce COVID risk^29,30^. We have termed this variable the “Big 2”, and it is coded as reporting masking and social distancing, reporting one of the two, or reporting neither.” |
| Methods: It is also unclear why the wide-ranging answers of good/fair/poor health status are combined together in a binary variable. Have the authors considered consistently using mean/median splits for binary classifications like these? | Self-reported health tends to assume a non-normal distribution with a mode around “excellent” and "very good”. We conceptually chose to dichotomize at this point to capture people who see some level illness when viewing their own health. |
| Results: It could be interesting to see whether the results hold in multivariate OLS regressions with standardized variables as well. Although logit regressions are indeed the most suitable approach for the given data, OLS regressions are often more intuitive to interpret. It is therefore (unofficially) a common practice to report both logit and OLS results in these cases, simply to facilitate the ease of reading. That being said, I leave this fully to the authors to decide whether this is something they would like to consider. | We agree that it is common to report coefficients from OLS regressions (linear probability models) for ease of interpretation. However, the linear probability model approach is most appropriate when the dependent variable is toward the center of the unit interval, where the logit regression is roughly linear. Our dependent variable has a mean of 0.118, which we view as on the margin of being appropriate for the LPM approach. We have therefore retained the logit reporting, but hope that the odds ratios we have reported are nevertheless relatively straightforward to interpret. |
| Discussion & Conclusions: The authors state that only two results are robust (having school-aged children and engaging in numerous mitigation behaviors), yet the paper stars with also mentioning age and income effects in the abstract. Please adjust the messaging to be consistent throughout the paper accordingly. (In fact, some other studies have shown the age effects to be in the other direction than the less-robust ones reported in this study, with younger people more likely to be infected and older people being more careful in this respect.) | We have updated our discussion at lines 208-211 to comment on all of our significant findings:  “Three observations were robust. First, our multi-variate regression found that older age groups were associated with higher odds of seropositivity and that living in a higher income household was associated lower odds of seropositivity. These findings further demonstrate socio-economic disparities in COVID-19 risk.” |
| Discussion and Conclusions: The sections seem to lack a discussion that puts the paper’s results into the broader context of the vast number of other related studies. How do the paper’s results add to the literature? How do the results relate to other studies? The readers of the paper would greatly benefit from more elaborate instructions for public health planners. What are the policy implications of the results? What should policy makers do (differently) after reading the paper? | We thank the reviewer for requesting a more detailed explanation of the policy implications for our findings. Being mindful of word count constraints, we’ve focused on the implications of our findings pertaining to having school-age children in the household and the personal and public health mitigation behaviors. Specifically, we’ve added the following regarding disambiguating the potential implications regarding the potential for transmission by school-aged children vs. effects caused by behaviors or attributes associated with having school-aged children (see lines 213-224):  “The significance and magnitude of the association between seropositivity and living with children of school age suggest that there are COVID-19 risk factors associated with the circumstances that accompany raising children. This may support the fact that school-age children can become infected and transmit SARS-CoV-2 infections and contribute to family and community spread; however, our observational findings cannot determine whether this is the specific driving force of our findings, or whether some other feature associated with having school-aged children made respondents to the survey more vulnerable to the spread of COVID-19. Further research should be conducted to determine if school-aged children are meaningful contributors to transmission, to inform whether broader testing efforts in schools might be a useful tool to identify infectious individuals, prevent outbreaks, and protect vulnerable members of the community.” |

**Additional Citations**

33.	Khanijahani A. Racial, ethnic, and socioeconomic disparities in confirmed COVID-19 cases and deaths in the United States: a county-level analysis as of November 2020. *https://doi.org/101080/1355785820201853067*. 2020;26(1):22-35. doi:10.1080/13557858.2020.1853067

34.	Khanijahani A, Iezadi S, Gholipour K, Azami-Aghdash S, Naghibi D. A systematic review of racial/ethnic and socioeconomic disparities in COVID-19. *Int J Equity Health*. 2021;20(1):1-30. doi:10.1186/S12939-021-01582-4/TABLES/2

35.	Krammer F, Srivastava K, Alshammary H, et al. Antibody Responses in Seropositive Persons after a Single Dose of SARS-CoV-2 mRNA Vaccine. *N Engl J Med*. 2021;384(14):1372-1374. doi:10.1056/NEJMC2101667/SUPPL_FILE/NEJMC2101667_DISCLOSURES.PDF

Detailed Information about the Minnesota COVID-19 Antibody Study (MCAS)

The following is more detailed information about this study in addition.

What is research?

Doctors and investigators are committed to your care and safety. There are important differences between research and treatment plans:

- The goal of research is to learn new things in order to help groups of people in the future. Investigators learn things by following the same plan with a number of participants, so they do not usually make changes to the plan for individual research participants. You, as an individual, may or may not be helped by volunteering for a research study.
- The goal of clinical care is to help you get better or to improve your quality of life. Doctors can make changes to your clinical care plan as needed.

Why am I being asked to take part in this research study?

We are asking you to take part in this research study because you or someone in your household are a resident of Minnesota who previously participated in this study with NORC at the University of Chicago (NORC). We are contacting your household again because you or someone in your household agreed to be reached for future follow-up studies.

What should I know about a research study?

- This page will explain this research study to you.
- Whether or not you take part is up to you.
- You can choose not to take part.
- You can agree to take part and later change your mind.
- Your decision will not be held against you.
- You can ask all the questions you want before you decide.

Why is this research being done?

The purpose of this study is to better understand the seroprevalence, or the presence of antibodies, to SARS-CoV-2, the virus that causes COVID-19, in Minnesota. Not everyone infected with SARS-CoV-2 has symptoms or knows they have been infected. Your participation is important because, by testing your blood for antibodies, we will learn more about the percentage of people in Minnesota who have antibodies against SARS-CoV-2.

Information learned from this study will help us better understand the spread of COVID-19 in Minnesota as well as potentially inform future public health recommendations and policy in Minnesota. NORC at the University of Chicago (NORC) and the University of Minnesota are conducting the study. This study is sponsored by Minnesota Department of Health.

How long will the research last?

It will take under 20 minutes to collect your blood sample and put it in the mail back to our lab.

What will I need to do to participate?

If you agree to participate, you will be asked to provide or confirm your mailing address and contact information. An antibody testing kit, which is a blood collection kit, will be sent to the address you provide. The antibody testing kit contains supplies to safely prick your finger and squeeze a few drops of blood into a collection device. The sample you will collect involves procedures similar to those conducted in a doctor’s office and have been carefully designed to minimize any risk to you. You will then send your blood sample back to our central lab in the pre-addressed prepaid return shipper that will be provided to you.

After you send in your sample we will send you a [$25/$50] gift card as well as your antibody test results to thank you for your time. More detailed information about the study procedures can be found under ***“What happens if I say yes, I want to be in this research?”***

Is there any way that being in this study could be bad for me?

Participation poses little or no risk. There may be minor discomfort that will resolve quickly after you prick your finger for a drop or two of blood. As with any research study, there is the potential risk of a breach of data confidentiality and subject privacy. However, the risk associated with this study is minimal as we store information on encrypted password protected devices. The data we are collecting cannot readily be used to directly identify an individual as we will be using unique alpha-numeric identifiers to label your information (as opposed to your name). Furthermore, we will be utilizing established guidelines and security measures at both NORC and the University of Minnesota to ensure the safety of research information.

More detailed information about the risks of this study can be found under ***“What are the risks of this study? and in the “What happens to the information collected for the research?” section***

Will being in this study help me in any way?

We cannot promise any benefits to you or others from your taking part in this research. However, possible benefits include knowledge of antibody test results (i.e. knowing if previously infected with SARS-CoV-2). In addition, society and investigators will benefit from the knowledge gained.

What happens if I do not want to be in this research?

You are free not to participate. If you choose not to participate, you will not receive a [$25/$50] gift card and you will not receive your antibody test results for SARS-CoV-2, the virus that causes COVID-19.

How many people will be studied?

We expect about 1600 people to be enrolled in this research study.

What happens if I say “Yes, I want to be in this research”?

Upon consent into the study, you will be asked to provide your mailing address to send your blood collection kits and a phone number to reach you. A blood collection kit with instructions for pricking your finger, self-collecting a blood spot sample, and sending the collected blood back to the lab where the antibody test will be conducted. You will be asked to register your name and your kit number in a secure web portal so that the test results will be communicated to you when completed. **This is a research test only and not a clinical test or a diagnosis from a doctor. If you have questions about your health or whether you have had COVID-19, talk with your doctor.**

What happens if I say “Yes”, but I change my mind later?

If you take part in this research study, and want to withdraw at a later date, you should tell us and we will have you withdrawn. Your choice not to be in this study will not negatively affect your right to any

present or future medical care or your ability to participate in future NORC or University of Minnesota-based studies. If you choose to be withdrawn from the study, we may contact you via phone to document reason for withdrawal. Data collected prior to withdrawal will still be used in data analysis unless you request otherwise. Following withdrawal, you will be removed from the active follow-up list to ensure that no additional follow-up contacts or data collection attempts are made.

Will it cost me anything to participate in this research study?

Taking part in this research study will not directly lead to any costs to you. The antibody test is provided free of charge.

What happens to the information collected for the research?

Efforts will be made to limit the use and disclosure of your personal information, including research study records, to people who have a need to review this information. We cannot promise complete confidentiality. Organizations that may inspect and copy your information include the Institutional Review Board (IRB), the committee that provides ethical and regulatory oversight of research, and other representatives of this institution, including those that have responsibilities for monitoring or ensuring compliance (such as the Quality Assurance Program of the Human Research Protection Program (HRPP)). Colleagues at Quansys Biosystems where the antibody tests will be performed will have access to your test result for the purpose of communicating results back to you and to our research team at the University of Minnesota.

The antibody test that we use is regulated by the Federal Drug Administration (FDA). As such, it is possible that the FDA may access the records, including your test results, from this study. Your test results from this study will not be connected to your personal medical records, however, and the FDA will have not have access to other personal or medical records as a result of this study.

Will my data be confidential?

NORC at the University of Chicago maintains the highest standards of confidentiality.
No information obtained in the course of this study may be disclosed in a manner in which the individual supplying the information is identifiable, except to a very small number of authorized staff on MCAS and at NORC, and then only for study administrative purposes, not for dissemination. Any violation of your confidentiality is subject to penalties of up to five years in prison and/or as much as a $250,000 fine.

How is my privacy protected?

All of your information is treated as confidential. Participation in MCAS is also entirely voluntary, and there are no penalties for not participating. A respondent's test results are never reported individually, but instead are grouped with results from other persons in the study to create statistical and analytical reports. In short, at no point does NORC or MCAS identify individual respondents and never publishes individual responses.

Will I receive research test results?

Yes. You will have access to your test results via a secure email to your inbox.

What will be done with my data and specimens when this study is over?

Your data and/or samples will not be used for any future research after this study is complete.

Will anyone besides the study team be at my consent meeting?

The risk associated with this study is minimal and there will not be an in-person consent meeting. The consent process will be performed on-line at the time and location of your choosing. If you wish to discuss the specifics of this research prior to signing informed consent, Dr. Beebe or a member of his research team will schedule a physically distanced visit, likely via phone or Zoom.

Whom do I contact if I have questions, concerns or feedback about my experience?

This research has been reviewed and approved by an IRB within the Human Research Protections Program (HRPP). To share feedback privately with the HRPP about your research experience, call the Research Participants’ Advocate Line at [612-625-1650](about:blank) (Toll Free: 1-888-224-8636) or go to [z.umn.edu/participants](about:blank). You are encouraged to contact the HRPP if:

- Your questions, concerns, or complaints are not being answered by the research team.
- You cannot reach the research team.
- You want to talk to someone besides the research team.
- You have questions about your rights as a research participant.
- You want to get information or provide input about this research.

Will I have a chance to provide feedback after the study is over?

The HRPP may ask you to complete a survey that asks about your experience as a research participant. You do not have to complete the survey if you do not want to. If you do choose to complete the survey, your responses will be anonymous.

If you are not asked to complete a survey, but you would like to share feedback, please contact the study team or the HRPP. See the “Investigator Contact Information” of this form for study team contact information and “Whom do I contact if I have questions, concerns or feedback about my experience?” of this form for HRPP contact information.

Can I be removed from the research?

While unlikely, the person in charge of the research study can remove you from the research study without your approval.

What happens if I am injured while participating in this research?

In the unlikely event that this research activity results in an injury, obtaining treatment will be your responsibility due to the fact that members of our study team will not be present during the finger prick. The cost of any treatment would be solely your responsibility. If you think that you have suffered a research related injury let the study principal investigator know right away.

Will I be compensated for my participation?

As a thank you for participating, we will send you a [$25/$50] gift card code.
